# A case of pancreatic adenosquamous cell carcinoma with a pseudocyst following curative surgery

**DOI:** 10.1186/s40792-024-01868-z

**Published:** 2024-04-01

**Authors:** Nao Kitasaki, Tomoyuki Abe, Masashi Inoue, Marino Teshima, Masataka Nakagawa, Masatoshi Kochi, Ryuichi Hotta, Kazuhiro Toyota

**Affiliations:** Higashihiroshima Medical Center, National Hospital Organization, Higashihiroshima, Japan

**Keywords:** Pancreatic cancer, Adenosquamous cell carcinoma, Distal pancreatectomy

## Abstract

**Background:**

Pancreatic adenosquamous cell carcinoma (PASC) is a relatively rare histological type of pancreatic malignancy, and preoperative diagnosis is difficult because of its rarity. PASC accounts for 1–4% of all pancreatic cancers, and even after curative surgery, its prognosis is poorer than that of ordinary pancreatic adenocarcinoma. Pathologically, it shows glandular and squamous differentiation of cells. Complete resection is the only method to achieve a good long-term prognosis, and an increasing doubling time of PASC is considered to indicate early recurrence after surgery. Here, we report a rare case of PASC with an infected pancreatic cyst that was difficult to treat, along with a review of the literature.

**Case presentation:**

A woman in her 80s with a history of breast cancer presented with pericardial pain. Computed tomography revealed a 20-mm hypovascular tumor in the body of the pancreas and a 27-mm pseudocyst. Endoscopic retrograde cholangiopancreatography showed a severe main pancreatic duct stenosis in the body of the pancreas that made cannulation impossible, and contrast media extravasation was due to pancreatic duct disruption in the pancreatic tail. Endoscopic fine-needle aspiration revealed that the tumor was a PASC. Because the patient had an infected pancreatic cyst, central intravenous nutrition and antibiotics were administered, which stabilized her general condition. She was diagnosed with resectable PASC and underwent distal pancreatectomy with lymphadenectomy. The postoperative course was uneventful. Immunohistochemical analysis of the resected specimen confirmed T2N0M0 stage IB. Systemic adjuvant chemotherapy with S-1 is ongoing.

**Conclusion:**

Appropriate preoperative management and preoperative accurate staging (T2N0M0 stage IB) of PASC with curative surgery can ensure predictable outcomes.

## Background

Pancreatic adenosquamous cell carcinoma (PASC) is a relatively rare histological type of pancreatic malignancy, and preoperative diagnosis is difficult because of its rarity. PASC accounts for 1–4% of all pancreatic cancers, and even after curative surgery, its prognosis is poorer than that of ordinary pancreatic adenocarcinoma [1–3]. Pathologically, it shows glandular and squamous differentiation of cells. Complete resection is the only method to achieve a good long-term prognosis, and an increasing doubling time of PASC is considered to indicate early recurrence after surgery.

However, the pathophysiology of PASC remains unclear. Several hypotheses regarding the histogenesis of PASC can be summarized as follows: adenocarcinoma transforms into squamous cell carcinoma (SCC), bipotential undifferentiated cell, collision tumor, or squamous metaplasia origin. Radiological findings are the key to disease diagnosis, and a precise preoperative diagnosis allows curative resection to be performed as early as possible [4, 5]. Here, we report a rare case of PASC with an infected pancreatic cyst that was difficult to treat, along with a review of the literature.

## Case presentation

**Fig. 1:**
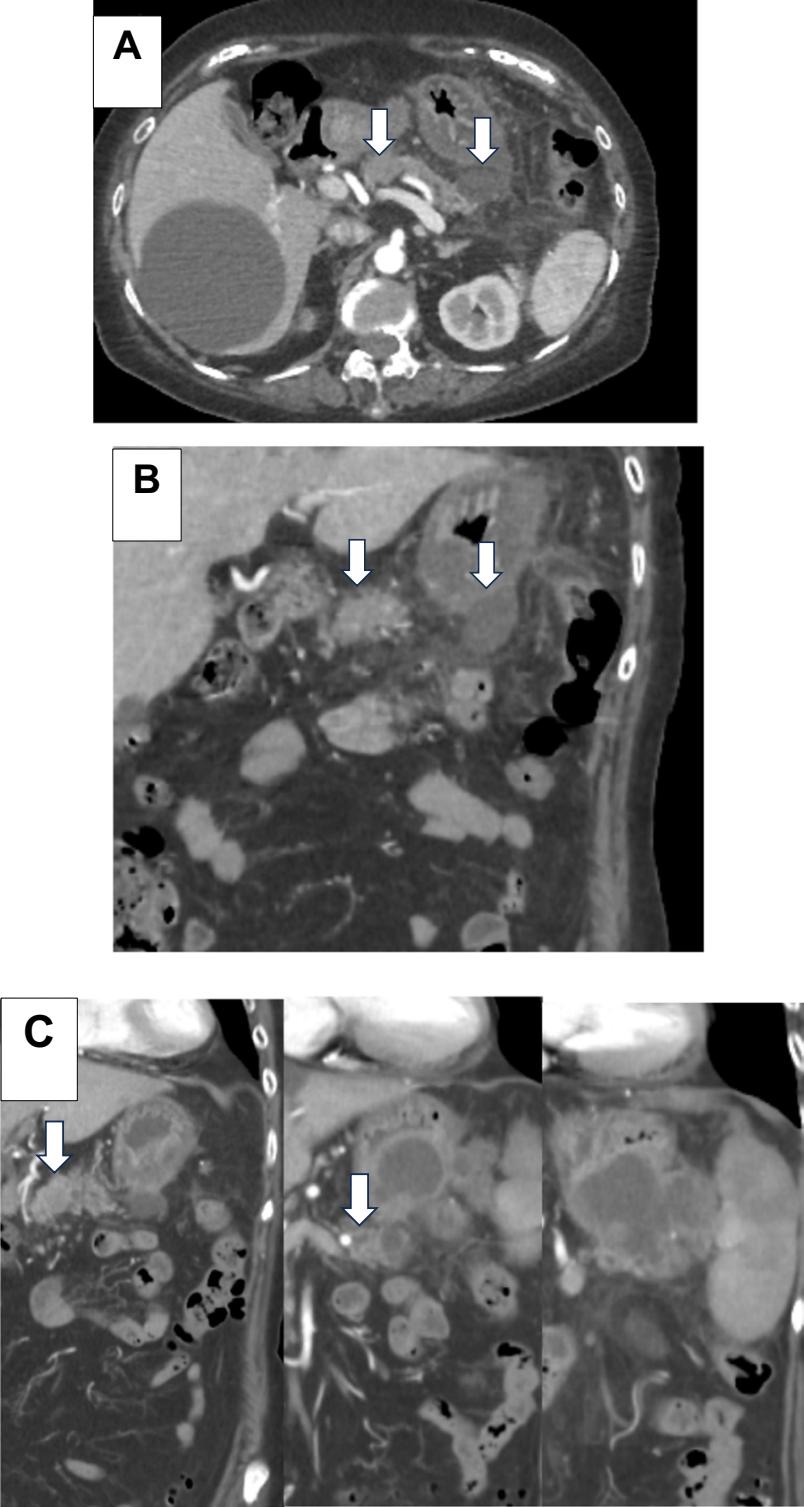
Abdominal dynamic CT findings. **A**–**C** CT revealed a 20-mm hypovascular tumor in the body of the pancreas and a 27-mm pseudocyst. The distal part of the main pancreatic duct was dilated due to obstruction by the tumor, and the pancreatic cyst was continuous with the pancreas (white arrow). *CT* computed tomography

**Fig. 2:**
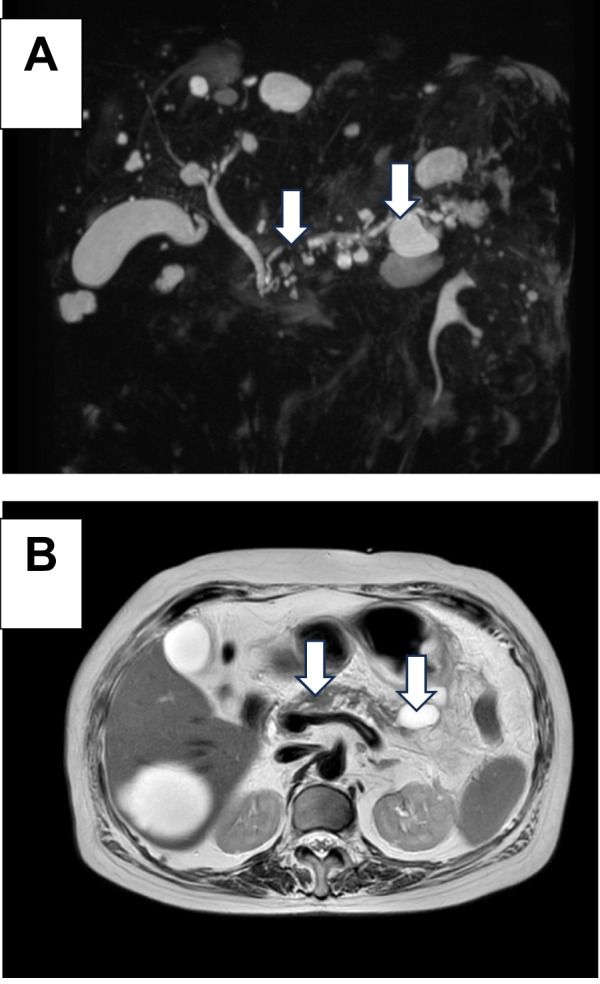
Magnetic resonance cholangiopancreatography findings. **A**, **B** Magnetic resonance cholangiopancreatography revealed a tumor in the pancreatic body and complete obstruction of ~ 12 mm of the main pancreatic duct. A high-signal pancreatic effusion and pseudocyst were seen on a T2-weighted image of the pancreatic tail (white arrow)

**Fig. 3:**
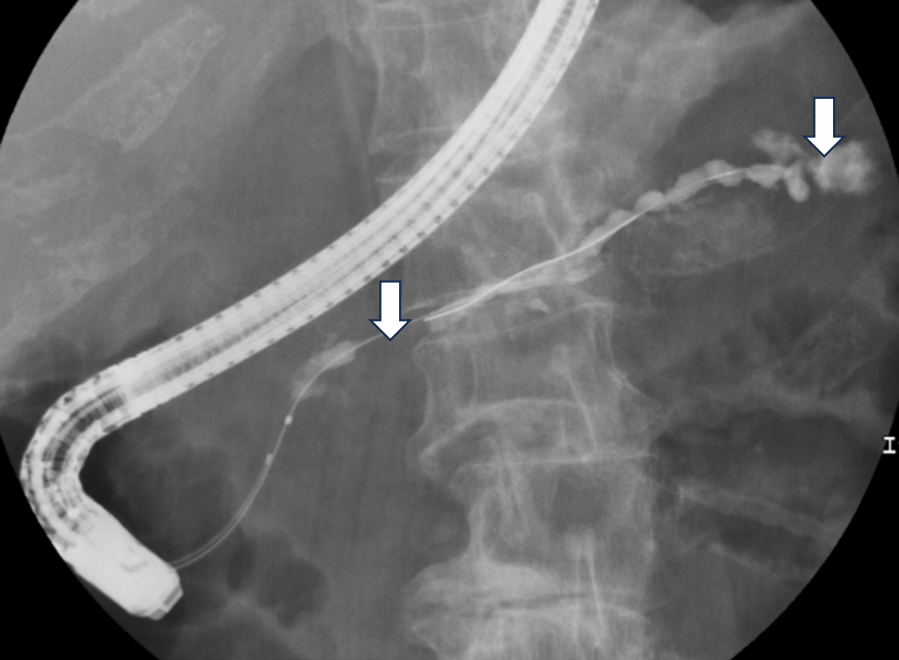
Endoscopic retrograde cholangiopancreatography findings. Endoscopic retrograde cholangiopancreatography showed that the main pancreatic duct stenosis in the body of the pancreas was so severe that cannulation was impossible, and extravasation of contrast media was due to pancreatic duct disruption in the pancreatic tail (white arrow)

A woman in her 80s presented to our hospital for a pericardial pain examination. Her medical history included breast cancer and dementia. Laboratory analysis demonstrated an elevated inflammatory response with pancreatic enzyme, normal carcinoembryonic antigen, and carbohydrate antigen 19-9 levels. However, elevated levels of duke pancreatic monoclonal antigen type 2 (DUPAN-2) were observed (454 U/mL). Computed tomography (CT) revealed a 20-mm hypovascular tumor in the body of the pancreas and a 27-mm pseudocyst. The distal part of the main pancreatic duct was dilated due to obstruction by the tumor, and the pancreatic cyst was continuous with the pancreas. A contrast effect was observed in the early phase without significant lymph node enlargement (Fig. 1A–C). Magnetic resonance cholangiopancreatography revealed a tumor in the pancreatic body and complete obstruction of approximately 12 mm of the main pancreatic duct. A high-signal pancreatic effusion and a pseudocyst were seen on a T2-weighted image of the pancreatic tail (Fig. 2A, B). Endoscopic retrograde cholangiopancreatography showed that the main pancreatic duct stenosis in the body of the pancreas was so severe that cannulation was impossible, and contrast media extravasation was due to pancreatic duct disruption in the pancreatic tail (Fig. 3) Endoscopic ultrasonography fine-needle aspiration revealed that the tumor was PASC.

*Aeromonas caviae* was detected in culture examination of the pancreatic fluid, and a diagnosis of an infected pancreatic cyst was made. Central intravenous nutrition and antibiotics were administered, which stabilized her general condition. The patient was diagnosed with resectable PASC and underwent distal pancreatectomy with splenectomy. We performed washing cytology, which revealed a negative result by frozen section analysis. The intraoperative duration was 280 min, and the blood loss was 600 mL.

**Fig. 4:**
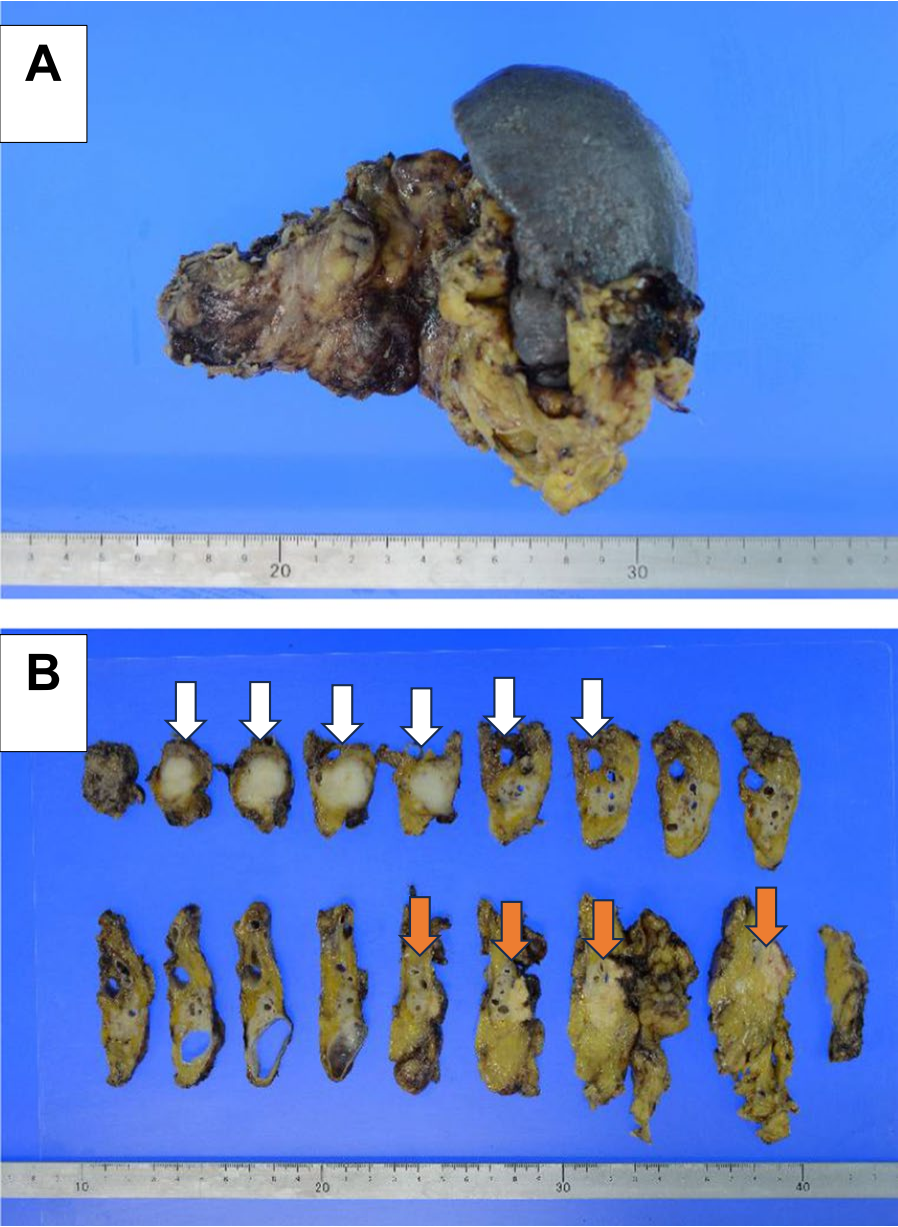
Histopathological findings of the resected specimen. **A**, **B** Macroscopically, a 25 × 22 × 22 mm lesion was observed in the body of the pancreas, showing complete occlusion of the main pancreatic duct. The pancreatic resection margins were negative, and no lymph node metastases were identified. The tumor caused dilation of the peripheral pancreatic ducts, but there was no tendency of invasion of the pancreatic ducts peripheral to the tumor (white arrow). The caudal pancreas shows evidence of chronic sclerosing pancreatitis. Yellow-cell granuloma formation is evident after pancreatic effusion (orange arrow)

**Fig. 5:**
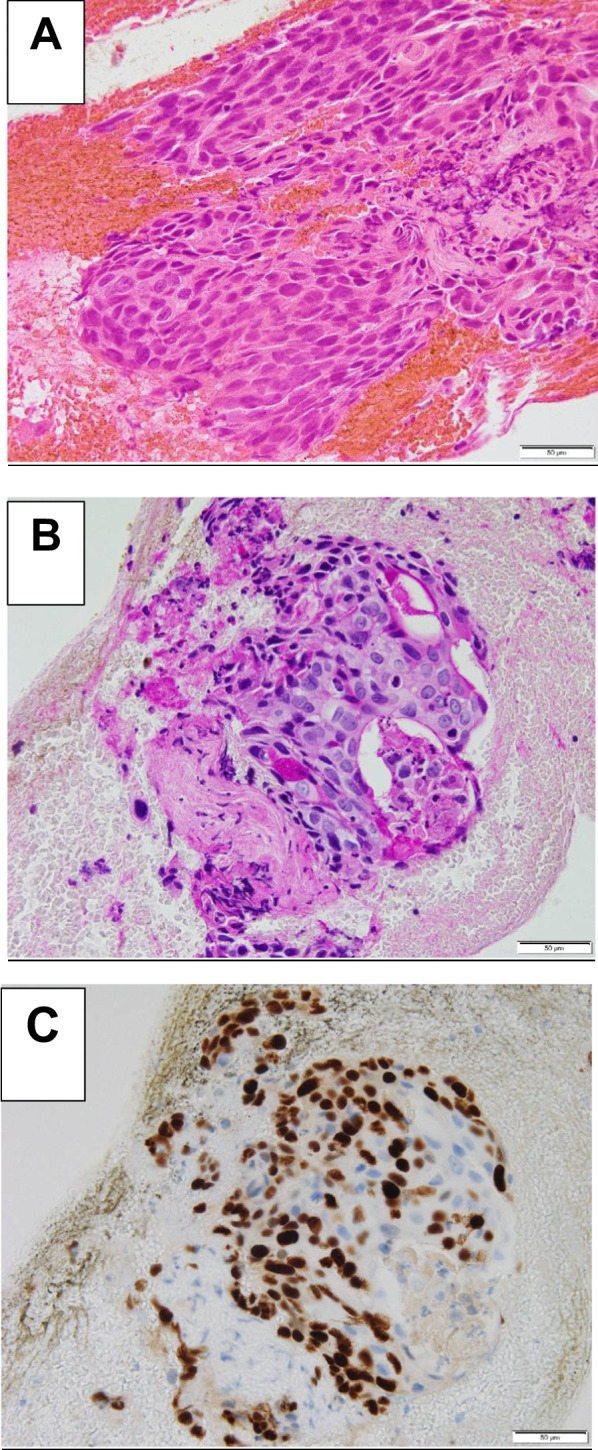
Histopathological findings of the resected specimen. **A** The cellular picture of adenocarcinoma is characterized by enlarged nucleoli, small and large immobile nuclei, and squamous cell carcinoma with enlarged nuclei and an oval shape. **B**, **C** Immunohistochemical analysis of the tumor showed that periodic acid–Schiff staining and anti-p40 immunostaining were positive, leading to the diagnosis of squamous cell carcinoma of the pancreatic gland

Macroscopically, a 25 × 22 × 22 mm lesion was observed in the body of the pancreas, indicating complete occlusion of the main pancreatic duct. The pancreatic resection margins were negative, and no lymph node metastases were identified. The caudal part of the pancreas showed evidence of chronic sclerosing pancreatitis. Yellow-cell granuloma formation was observed after pancreatic effusion (Fig. 4A, B). The cellular picture of adenocarcinoma was characterized by enlarged nucleoli, small and large immobile nuclei, and SCC with enlarged nuclei and an oval shape. Immunohistochemical analysis of the tumor showed that periodic acid–Schiff (PAS) staining and anti-p40 immunostaining were positive, leading to the diagnosis of SCC of the pancreatic gland (Fig. 5A–C).

The postoperative course was uneventful. Immunohistochemical analysis of the resected specimen confirmed the diagnosis of tumor node metastasis [TNM] classification as T2N0M0 stage IB. Systemic adjuvant chemotherapy with S-1 is ongoing without any recurrence.

Written informed consent was obtained from the patient for the publication of this case report and accompanying images.

## Discussion

PASC shows an aggressive behavior, and its prognosis, even after curative surgery, remains poorer than that of conventional pancreatic ductal adenocarcinoma (PDAC). Boecker et al. reported that the median overall survival in PASC was worse than in PDAC (8.2 months vs. 20.1 months, *P* = 0.089) [3]. Curative surgery significantly affects the prognosis of patients with PASC and PDAC. Positive lymph nodes, margin-positive resections, and older age (≥ 65 years) were associated with a poor prognosis in patients with PASC [5–7]. Perioperative systemic chemotherapy combined with pancreatectomy is the standard treatment for resectable and borderline PDAC. However, the optimal perioperative systemic chemotherapy for PASC remains unknown [5, 8]. Wild et al. reported that a platinum-based agent used as a component of adjuvant therapy prolongs recurrence-free survival in patients with PASC [8]. Notably, patients with PASC who are at high risk of recurrences, such as those with a large tumor diameter, advanced T stage, and tumor differentiation, tend to show high sensitivity to adjuvant chemotherapy [5]. In the genetic profile of PASC, *PTEN* was significantly more altered than in that of PDAC, indicating that PI3K inhibitors could play an important role in the antitumor effect in the future [9].

Despite the unavailability of a well-established perioperative systemic chemotherapy regimen for patients with PASC, curative surgery following adjuvant chemotherapy may prolong survival, as observed in our case. Moreover, Aigner et al. reported the effectiveness of intra-arterial infusion chemotherapy and isolated upper abdominal perfusion chemotherapy [10]. Nomogram-based models for evaluating personalized long-term prognosis in patients with PASC were retrieved from the Surveillance, Epidemiology, and End Results program database [11]. Considering the limited data regarding optimal regimens related to PASC, this nomogram may help select patients who would gain the maximum benefit of the treatment. Therefore, further studies are required in adjuvant chemotherapy for PASC.

The malignant potential of PASC is strongly associated with the tumor’s SCC components. Adenosquamous cell carcinomas originating from organs lined by glandular epithelium, such as the colon, rectum, breast, and prostate, are rare. Their clinical prognosis is poor, and their behavior is more aggressive than that of conventional adenocarcinomas. Several studies have reported significantly shorter doubling times for SCC than for lung adenocarcinomas, and some studies have reported that PASC tumors are larger than PDAC lesions. The pathogenesis of PASC remains unclear, although the following hypotheses have been proposed: (a) it originates from cancer stem cells, followed by (b) squamous metaplasia of the intestinal mucosa, (c) malignant squamous metaplastic transformation of adenocarcinoma, and (d) collision of the two components.

The cancer stem cell hypothesis is considered to be the most likely pathogenic contributor, as reported in previous studies [12, 13]. However, the association between the number of SCC components and the prognosis remains unclear. Voong et al. reported that the percentage of squamous differentiation was not associated with median overall survival (< 30% vs. ≥ 30%) [5]. Conversely, some studies have reported that progression patterns of the SCC component are characterized by vascular invasion as opposed to metastasis to the surrounding lymph nodes, which may explain the greater tendency of PASC to show vascular invasion and distant metastasis to the lungs and liver compared to that of PDAC [14, 15]. It has been hypothesized that the proliferation of the SCC component of PASC results in distant metastasis to the lungs and liver and is therefore associated with a poorer prognosis than that of PDAC.

The rarity of pancreatic PASC and the rapidity of its progression explain the difficulty in diagnosing this malignancy. In this case, the imaging findings led to an initial suspicion of a tumor, and various tests were performed to confirm the diagnosis. Accordingly, early treatment of the infected pancreatic cyst and radical surgery for the advanced PASC were possible, rendering this case to be a useful example for determining a treatment strategy. Clinically, anorexia and weight loss due to abdominal pain, with or without jaundice, are typical initial symptoms similar to those used to diagnose primary pancreatic adenocarcinoma [1]. Diagnosis is based on CT and endoscopic ultrasound biopsy in the absence of metastatic sites, as in our case report. CT of PASC lesions commonly shows the presence of central necrosis within the tumor mass and the propensity for vascular and nerve encasement [16].

To our knowledge, endoscopic ultrasound features have rarely been described in the literature, and PASC usually appears as a solid and hypoechoic lesion that is not well-defined [17]. This case is unique in that it was discovered because of the appearance of symptoms due to pancreatic fluid leakage caused by pancreatic duct obstruction. Normally, pancreatic cancer is characterized by atrophy of the peripheral pancreas due to pancreatic duct obstruction. However, PASC is characterized by dilatation and intratumoral necrosis, resulting in duct dilation and collapse [18, 19]. In squamous epithelium-dominant tumors, the central part of the tumor may undergo necrosis because angiogenesis cannot keep pace with the tumor, or the interstitial space may become wider because of the tendency toward keratinization, which may contribute to the formation of cysts [20]. In cases of mass formation resulting in pancreatic duct dilation, it is essential for diagnosis and treatment. PASC is a rare histological type of pancreatic cancer with a poorer prognosis than conventional PDAC. Special attention should be paid to early recurrence of PASC and delayed remnant pancreatic cancer.

This case report has been reported in line with the SCARE Criteria.

## Conclusions

We presented a case of PASC diagnosis requiring CT, endoscopic retrograde cholangiopancreatography, and fine-needle aspiration due to its atypical presentation with pericardial pain. Prompt intervention with central intravenous nutrition and antibiotics stabilized the patient with an infected pancreatic cyst, enabling a subsequent successful distal pancreatectomy. Preoperative accurate staging (T2N0M0 stage IB) with curative surgery led to a predictable outcome.

## Data Availability

Data sharing not applicable to this article as no datasets were generated or analyzed during the current study.

## Abbreviations

PASC: Pancreatic adenosquamous cell carcinoma
DUPAN-2: Duke pancreatic monoclonal antigen type 2
PAS: Periodic acid–Schiff
PDAC: Pancreatic ductal adenocarcinoma
SCC: Squamous cell carcinoma
CT: Computed tomography
